# Safety and tolerability of CFI-400945, a first-in-class, selective PLK4 inhibitor in advanced solid tumours: a phase 1 dose-escalation trial

**DOI:** 10.1038/s41416-019-0517-3

**Published:** 2019-07-15

**Authors:** Zachary W. Veitch, David W. Cescon, Trisha Denny, Lisa-Maria Yonemoto, Graham Fletcher, Richard Brokx, Peter Sampson, Sze-Wan Li, Trevor J. Pugh, Jeffrey Bruce, Mark R. Bray, Dennis J. Slamon, Tak W. Mak, Zev A. Wainberg, Philippe L. Bedard

**Affiliations:** 1 Division of Medical Oncology and Hematology, Department of Medicine, Princess Margaret Cancer Centre, University Health Network, Toronto, ON Canada; 2 Campbell Family Institute for Breast Cancer Research, Princess Margaret Cancer Centre, University Health Network, Toronto, ON Canada; 3 University of California Los Angeles David Geffen School of Medicine, Los Angeles, CA USA; 4 Princess Margaret Cancer Centre, University Health Network, Toronto, ON Canada

**Keywords:** Drug development, Targeted therapies

## Abstract

**Background:**

CFI-400945 is a first-in-class oral inhibitor of polo-like kinase 4 (PLK4) that regulates centriole duplication. Primary objectives of this first-in-human phase 1 trial were to establish the safety and tolerability of CFI-400945 in patients with advanced solid tumours. Secondary objectives included pharmacokinetics, pharmacodynamics, efficacy, and recommended phase 2 dose (RP2D).

**Methods:**

Continuous daily oral dosing of CFI-400945 was evaluated using a 3+3 design guided by incidence of dose-limiting toxicities (DLTs) in the first 28-day cycle. Safety was assessed by CTCAE v4.0. ORR and CBR were evaluated using RECIST v1.1.

**Results:**

Forty-three patients were treated in dose escalation from 3 to 96 mg/day, and 9 were treated in 64 mg dose expansion. After DLT occurred at 96 and 72 mg, 64 mg was established as the RP2D. Neutropenia was a common high-grade (19%) treatment-related adverse event at ≥ 64 mg. Half-life of CFI-400945 was 9 h, with *C*_max_ achieved 2–4 h following dosing. One PR (45 cycles, ongoing) and two SD ≥ 6 months were observed (ORR = 2%; CBR = 6%).

**Conclusions:**

CFI-400945 is well tolerated at 64 mg with dose-dependent neutropenia. Favourable pharmacokinetic profiles were achieved with daily dosing. Response rates were low without biomarker pre-selection. Disease-specific and combination studies are ongoing.

**Trial Registration:**

Clinical Trials Registration Number – NCT01954316 (Oct 1st, 2013)

## Background

The polo-like kinases (PLK) are a family of serine/threonine kinases that regulate mitotic entry, DNA repair, and stress responses in proliferating cells.^1,2^ Polo-like kinase 4 (PLK4/SAK) is an atypical family member sharing 37% similarity with PLK1, a key driver of mitotic entry and the subject of multiple drug development efforts.^3^ As an upstream regulator of centriole duplication,^4^ PLK4 is aberrantly expressed in a variety of malignancies and its expression has been associated with adverse survival outcomes in solid^5–9^ and haematologic malignancies.^10^ PLK4 was identified as a therapeutic target through a kinome-wide short interfering RNA (siRNA) screen,^11^ and functional genomic studies have confirmed it as an essential cell survival gene.^12–14^ Depletion of PLK4 in cancer cells by siRNA prevents centriole duplication, causing mitotic defects and cell death.^11^

CFI-400945 is a first-in-class, oral inhibitor of PLK4 identified through an academic drug discovery programme at the Campbell Family Institute (CFI) for Breast Cancer Research, at the University Health Network in Toronto, Canada. CFI-400945 inhibition is both potent (IC_50_ = 2.8 nM) and selective for PLK4 (>1000-fold) relative to other PLK family members (PLK1, PLK2, PLK3; IC_50_ > 50 μM). Only nine other protein kinases (Abl [T315I], TrkA/B, Bmx, FGFR1/2, Tie2, Ros, AurB) are inhibited by CFI-400945 with an IC_50_ < 100 nM. Preclinical studies have demonstrated robust in vivo antitumour activity of CFI-400945, including in patient-derived xenograft (PDX) models across multiple tumour types.^11,15,16^ Further preclinical testing of CFI-400945 metabolism has identified it as an inducer of cytochrome P450 (CYP) enzyme 1A2, a time-dependent inhibitor of CYP1A2 and 3A4, and also an inhibitor of drug efflux transporters P-glycoprotein, BCRP, BSEP, OCT1, OCT2, and OATP1B3. Toxicology studies in rodents and dogs have shown haematopoietic effects (myeloid suppression, bone marrow hypocellularity) as primary toxicities.

Based on its preclinical activity, this first-in-class, first-in-human phase 1 clinical trial (NCT01954316) was designed to evaluate the safety, pharmacokinetics (PKs), pharmacodynamics, recommended phase 2 dose (RP2D), and preliminary clinical antitumour activity of CFI-400945 administered orally to patients with advanced solid tumours.

## Patients and methods

### Population

Patients were consented and enrolled at two academic cancer centres (Princess Margaret Cancer Centre, Toronto, Canada, and University of California Los Angeles, Los Angeles, USA) prior to initiation of study procedures. Eligible patients were aged ≥18 years with histologically confirmed advanced cancer that had progressed; for which no standard anticancer therapy option was available; had an Eastern Cooperative Oncology Group (ECOG) performance status of 0–1; had measurable disease by RECIST v 1.1;^17^ and had not received granulocyte colony-stimulating factor (G-CSF) within 14 days of first dose of study drug. Further eligibility criteria can be found in Supplementary Information 1a.

This study protocol (NCT01954316) was approved by local institutional review boards (Princess Margaret Cancer Centre - Ontario Cancer Research Ethics Board [OCREB] - #13-050; OHRPP – Office of the Human Research Protection Program - #13-001872) of the two participating institutions and adhered to Good Clinical Practice guidelines, the Declaration of Helsinki, and local laws. All patients provided written informed consent prior to initiation of study procedures.

### Study design and objectives

The primary objective of this two-centre, open-label, phase 1 dose-escalation and expansion study were to assess the safety and tolerability of CFI-400945 oral tablet formulation (1.5, 8, or 32 mg strengths), given once daily on a continuous dosing schedule. Secondary objectives of this study were to assess the PKs, pharmacodynamics, and clinical benefit of CFI-400945. A standard 3+3 dose-escalation scheme was used to identify the maximally tolerated dose (MTD) and RP2D. Three to six evaluable patients per treatment cohort were assigned to receive CFI-400945 continuously in 28-day treatment cycles. Dosing occurred at least 2 h after, and 1 h before, any food (i.e. fasting) at dose levels ranging from 3 to 96 mg (Supplementary Information 1b).

Once the RP2D was defined for dose expansion, the protocol was amended to include a run-in period of 7 days of continuous dosing followed by a 7-day treatment-free interval (Cycle 0; 14 days total) to identify patients at risk of early, high-grade neutropenic events. Patients without evidence of high-grade neutropenia during this run-in period resumed daily dosing in standard 28-day cycles. Patients received CFI-400945 until disease progression, unacceptable toxicity, patient withdrawal, or investigator-recommended withdrawal from treatment.

### MTD, dose-limiting toxicity (DLT), and RP2D

DLT were assessed during the first 28 days of treatment (Cycle 1). Following a protocol amendment, DLT were assessed for patients in the expansion cohort during the first 42 days (14-day run-in [Cycle 0] and 28-day Cycle 1).

Select haematologic DLT included febrile neutropenia grade ≥3 and/or neutropenia grade ≥3 associated with infection or asymptomatic neutropenia grade ≥3 that persisted for >7 days. Use of haematopoietic growth factors (e.g. G-CSF, erythropoietin) in Cycle 1 was not permitted unless a DLT occurred. Usage beyond Cycle 1 was not permitted, except for short-term G-CSF to treat neutropenic complications in patients otherwise benefitting from CFI-400945 following approval by the Medical Monitor. Select non-haematologic DLT included failure to receive ≥21 of the 28 (75%) planned doses by the end of Cycle 1 or delay in starting Cycle 2 by >14 days due to drug-related toxicity. Additional DLT information is available in Supplementary Information 1c.

The RP2D was based on the MTD established during dose escalation together with a comprehensive review of toxicities, PKs, and efficacy outcomes.

### Safety assessments

All patients who received at least one dose of CFI-400945 were included in analyses of the safety-evaluable population. Data cut-off for the safety-evaluable population was June 5, 2018. Adverse events (AEs) were assessed according to the National Cancer Institute (NCI) Common Terminology Criteria for Adverse Events (CTCAE) v4.03. Investigators determined the causal relationship of an AE as either unrelated, possibly, probably, or related to study drug. Further information is available in Supplementary Information 1d.

### PK analyses

Plasma PKs of CFI-400945 was determined for all patients enrolled in the study who received at least 1 dose of study drug and had evaluable PK samples. Serial blood samples for determination of plasma levels of CFI-400945 were collected for each dose level on Days 1 and 28 of Cycle 1. In the expansion cohort, blood samples were collected on Day 1 for Cycle 0 and Day 28 of Cycle 1. Additional samples were drawn prior to dosing on Day 15 of subsequent cycles.

Samples were analysed in accordance with all applicable United States Federal Drug Administration (FDA) and Organisation for Economic Co-operation and Development (OECD) regulations. Human plasma samples containing CFI-400945 were collected with K_3_-EDTA as the anticoagulant. CFI-400945 ^13^C_6_ was added as the internal standard and samples were prepared by liquid/liquid extraction. After evaporation of the extraction solvent, sample extracts were reconstituted and analysed by reversed-phase high-performance liquid chromatography using a Waters Atlantis dC18 column. The mobile phase was nebulised using heated nitrogen in a Z-spray source/interface set to electrospray positive ionisation. The ionised compounds were detected using mass spectrometry. The lower limit of quantification was 20 pg/ml. Samples were analysed at Microconstants, San Diego, CA. PK parameters (i.e. *C*_max_, AUC_0–24 h_, *t*_1/2_) were calculated using “PK Functions for Microsoft Excel” provided by Usansky et al. (https://www.pharmpk.com/soft.html).

### Efficacy assessments and correlative studies

Diagnostic imaging response assessments (computed tomography or magnetic resonance imaging) were performed at baseline and at the end of every two cycles or as clinically indicated. All patients who had at least one post-baseline radiographic assessment of target, non-target, and new lesions constituted the efficacy-evaluable population. Patients were evaluated for complete response (CR), partial response (PR), stable disease (SD) ≥6 months, or progressive disease as determined by RECIST v1.1.^17^ Objective response rate (ORR = CR + PR) and clinical benefit rate (CBR = CR + PR + SD ≥ 6 months) were evaluated. Data cut-off for the efficacy-evaluable population was January 21, 2019.

Archival tissue was requested for all participants, and optional image-guided fresh tumour core needle biopsies for patients in the expansion cohort were obtained at baseline, end of Cycle 1, and upon confirmation of radiological disease progression for patients who had initial response of CR, PR, or SD >4 months. For archival material, nucleic acids were extracted from formalin-fixed slides. Fresh biopsies were snap frozen or embedded in formalin, and immunohistochemistry was performed on formalin-fixed slides. Whole-exome sequencing (WES) was performed using a modified protocol based on the KAPA Hyper Prep Kit, prior to capture using a modified Agilent XT V6 + COSMIC exome workflow. Tumour DNA was sequenced to a depth of 250× coverage on Illumina NovaSeq platform. All libraries were validated on the MiSeq platform prior to deep sequencing. A post-progression biopsy was obtained from one patient as part of a separate research protocol (NCT02732860). Targeted sequencing (555 genes) was performed on this sample and exome sequencing was performed on a patient-derived xenograft (PDX) generated from this sample. Mutations were compared between primary, progression, and progression PDX in order to identify functional variants arising post-treatment.

## Results

### Patient characteristics

Fifty-six patients were enrolled between March 3, 2014 and January 21, 2019: 46 in dose escalation and 10 in dose expansion. Of these, 52 patients received at least 1 dose of study drug (43 in dose escalation and 9 in dose expansion). One patient remains on study treatment. Median age of the treated patients was 60 years (range 31–78) with a median body mass index of 23.0 kg/m^2^ (range 17.2–41.9). The majority were female (60%), had an ECOG performance status of 1 (52%), and were Caucasian (88%). Patients were heavily pretreated, with 63% having ≥3 prior lines of systemic treatment. Common tumour types included colorectal (29%), pancreatic (12%), biliary (10%), and breast (8%) (Table 1).

**Table 1:** Patient characteristics

Baseline characteristics	(*N* = 52)
Age, years	
Median (range)	60 (31–78)
Sex, *n* (%)	
Male	21 (40)
Female	31 (60)
ECOG performance status, *n* (%)	
0	25 (48)
1	27 (52)
Ethnicity, *n* (%)	
Caucasian	46 (88)
Other	6 (12)
BMI (kg/m^2^)	
Median (range)	23.0 (17.2–41.9)
Prior systemic therapies, *n* (%)	
0–1	5 (10)
2	14 (27)
≥3	33 (63)
Primary cancer type, *n* (%)	
Colorectal	15 (29)
Pancreatic	6 (12)
Biliary	5 (10)
Breast	4 (8)
Adenoid cystic	3 (6)
Appendiceal	3 (6)
Endometrial	3 (6)
Neuroendocrine	2 (4)
NSCLC	2 (4)
Prostate	2 (4)
Other^a^	7 (13)

*NSCLC* non-small cell lung cancer, *ECOG* Eastern Cooperative Oncology Group, *BMI* body mass index

^a^Other includes: adrenocortical, cervical, fallopian, hepatocellular, hemangiopericytoma, small bowel, and small cell carcinoma(s)

### Dose escalation, DLTs, MTD, and RP2D

Overall, 48 (92%) of the 52 patients were DLT evaluable (Table 2). Three patients did not complete the DLT period due to progressive disease or non-treatment-related AE and were replaced (Supplementary Fig. 1). One patient (72 mg) was considered DLT unevaluable following the administration of G-CSF after 1 day of grade 4 neutropenia (precluding assessment of duration). Eight dose levels from 3 to 72 mg/day were completed without a DLT. At 96 mg, one DLT (grade 3 febrile neutropenia) occurred. A second patient treated at 96 mg experienced grade 4 neutropenia lasting <7 days (6 days total) in Cycle 1. Although not a DLT, this required dose reduction to 72 mg because of recurrent grade 4 neutropenia after restarting CFI-400945 at 96 mg in Cycle 2 (outside of DLT window). Based on these AEs, 96 mg was considered intolerable and three additional patients (7 total) were evaluated at 72 mg. Two patients experienced DLT events in Cycle 1: one with asymptomatic grade 4 lipase and grade 3 amylase elevation, and the other with grade 3 neutropenia >7 days. Following these DLTs, an additional intermediate dose level of 64 mg was subsequently evaluated, in which no DLT events were observed in 6 patients.

**Table 2:** Evaluation of dose-limiting toxicities by dose level and description (*n* = 48)

CFI-400945 oral daily dosage											
Grade	3 mg (*n* = 3)	6 mg (*n* = 3)	11 mg (*n* = 3)	16 mg (*n* = 3)	24 mg (*n* = 3)	32 mg (*n* = 3)	48 mg (*n* = 3)	64 mg (*n* = 15)	72 mg^a^ (*n* = 6)	96 mg (*n* = 6)	DLT description
1	0	0	0	0	0	0	0	0	0	0	0
2	0	0	0	0	0	0	0	0	0	0	0
3	0	0	0	0	0	0	0	0	1 (16.7)	1 (16.7)	Neutropenia, febrile neutropenia
4	0	0	0	0	0	0	0	1 (6.7)	1 (16.7)	0	Febrile neutropenia, lipase elevated
5	0	0	0	0	0	0	0	0	0	0	0
Total number of patients with DLT at each dosage	0	0	0	0	0	0	0	1	2	1	4

*DLT* dose-limiting toxicity

^a^One patient removed, not DLT evaluable

Thus, 64 mg was selected as the RP2D for expansion and 9 additional patients were treated. One patient experienced grade 4 febrile neutropenia on Cycle 1 Day 15, with grade 4 neutropenia persisting for 9 days despite G-CSF. After review, the protocol was amended to include the Cycle 0 run-in and additional haematologic monitoring in Cycle 1. Three additional patients were enrolled without DLT events.

### Safety and tolerability

In the safety-evaluable population (*n* = 52), the median number of cycles administered was 2 (range 1–45). Common treatment-related AE occurring in >5% of patients were fatigue (37%), nausea (29%), diarrhoea (21%), neutropenia (21%), anorexia (19%), vomiting (8%), dyspepsia (6%), hypomagnesaemia (6%), and dehydration (6%) (Table 3). With the exception of neutropenia, these were generally low grade and showed no clear dose-dependent trends. Neutropenia was the most common grade 3/4 treatment-related AE (10/52; 19%), of which high-grade neutropenia was observed only in patients treated at ≥64 mg/daily. Only one patient experienced grade 2 neutropenia at the 48 mg dose level. Most (60%; 6/10) grade 3/4 neutropenic events occurred in Cycle 1, with a median time to first grade 3/4 neutropenic event of 18 days (range 14–126). The median time for neutrophils to recover to grade ≤1 was 7 days (range 6–14). Overall, 27 patients (52%) had dose interruptions, and 3 patients (6%) were dose reduced at the 72 mg (one patient reduced to 48 mg) and 96 mg (two patients reduced to 72 mg) dose levels for recurrent neutropenia. No patients treated at 64 mg were dose reduced. Treatment-related AEs leading to study discontinuation occurred in two patients, one with a DLT at 72 mg (grade 4 lipase) and another with a DLT in the 64 mg cohort (grade 3 febrile neutropenia). Long-term tolerability was demonstrated by one patient who remained on 48 mg at data cut-off for >3 years.

**Table 3:** Treatment-related adverse events (trAEs) occurring ≥5% by the number of patients per dose range for CFI-400945

Dosing cohort	3–32 mg cohort (*n* = 21)		48–64 mg cohort (*n* = 18)		72–96 mg cohort (*n* = 13)		3–96 mg cohort (*n* = 52)
Events	Grade 1/2, *N* (%)	Grade 3/4, *N* (%)	Grade 1/2, *N* (%)	Grade 3/4, *N* (%)	Grade 1/2, *N* (%)	Grade 3/4, *N* (%)	All trAEs, *N* (%)
Fatigue	5 (24)	0 (0)	9 (50)	0 (0)	5 (38)	0 (0)	19 (37)
Nausea	3 (14)	0 (0)	5 (28)	0 (0)	6 (46)	0 (0)	14 (27)
Diarrhoea	4 (19)	0 (0)	3 (17)	1 (6)	3 (23)	0 (0)	11 (21)
Neutropenia	0 (0)	0 (0)	1 (6)	3 (17)	0 (0)	7 (54)	11 (21)
Anorexia	4 (19)	0 (0)	2 (11)	0 (0)	4 (31)	0 (0)	10 (19)
Vomiting	1 (5)	0 (0)	1 (6)	1 (6)	1 (8)	0 (0)	4 (8)
Dyspepsia	1 (5)	0 (0)	1 (6)	0 (0)	1 (8)	0 (0)	3 (6)
Dehydration	1 (5)	0 (0)	1 (6)	0 (0)	1 (8)	0 (0)	3 (6)
Hypomagnesaemia	0 (0)	0 (0)	1 (6)	0 (0)	2 (15)	0 (0)	3 (6)

### Pharmacokinetics

**Fig. 1:**
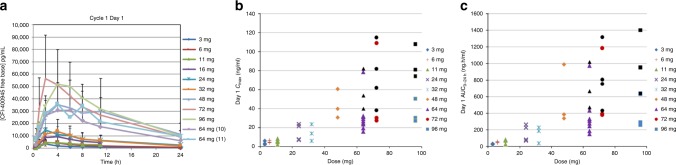
Pharmacokinetics of CFI-400945. Plasma concentration of CFI-400945 as a function of time and dose level following dosing on Cycle 1 day 1 (**a**). Cmax (**b**) and AUC (**c**) as a function of dose level following dosing on day 1 are also shown. Shapes represented in black (**b**/**c**) indicate patients experiencing grade 3 or 4 neutropenia at various dose levels *C*_max_ concentration maximum. *AUC* area under the curve

PK analyses of CFI-400945 by dose level following a single dose are shown in Fig. 1. Following oral administration of CFI-400945 in a fasting state, plasma concentrations rose rapidly to a maximum concentration (*C*_max_) generally 2–4 h after dosing (Fig. 1a). For patients treated with 64 mg on Day 1, the average *C*_max_ was 37 ng/ml with an AUC_0–24 h_ of 428 ng × h/ml and a half-life (t_1/2_) of 9 h. Across all dose levels, exposure at Day 28 was on average 37% higher than exposure at Day 1, which is consistent with the trough plasma levels of CFI-400945 that were present prior to the patient taking their Day 28 dose. For the patients treated at 64 mg, after 28 days of dosing the average *C*_max_ was 44 ng/ml and the AUC_0–24 h_ was 564 ng × h/ml. CFI-400945 exposure was generally dose proportional with increasing *C*_max_ and AUC_0–24 h_ values with increasing dose (*R*^2^ values of 0.90 and 0.88, respectively [Supplementary Fig. 2]). Grade 3/4 neutropenia was associated with AUC_0–24 h_ values >400 ng × h/ml (Fig. 1b) and *C*_max_ values ≥38 ng/ml (Fig. 1c).

### Efficacy evaluation

**Fig. 2:**
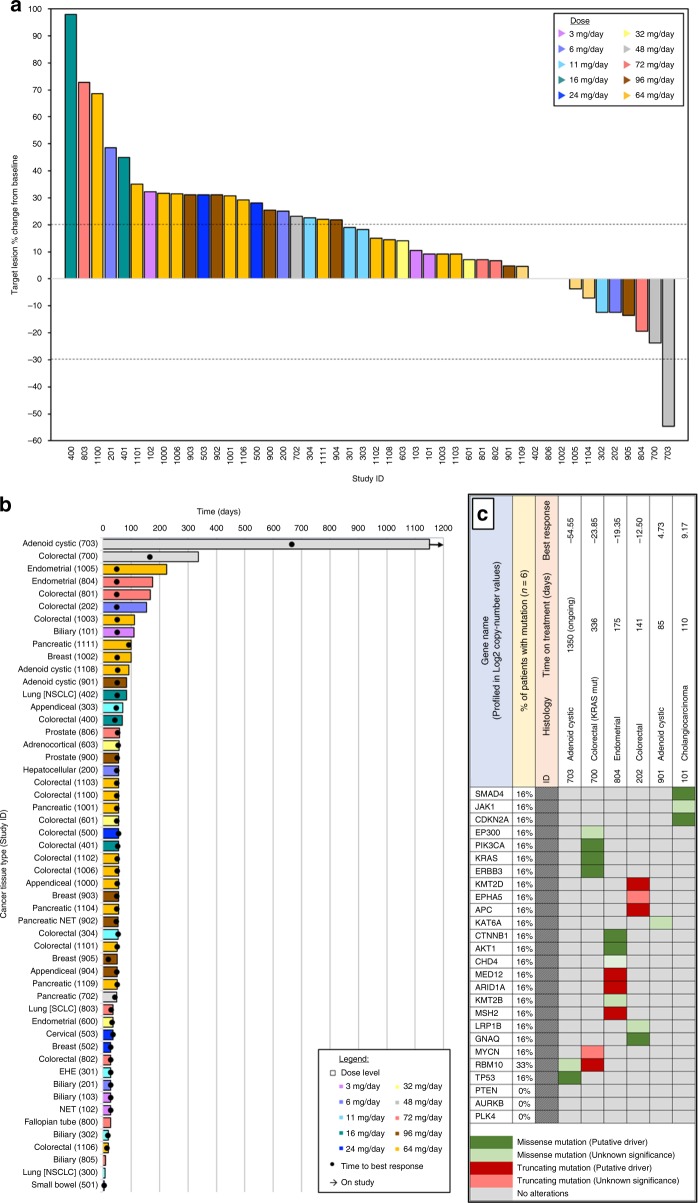
Waterfall plot (**a**) of individual patient target lesion best response, swimmers plot (**b**) of time on study by tissue type, and select patient oncoprint heatmap (**c**) by whole-exome sequencing. *NSCLC* non-small cell lung carcinoma, *NET* neuroendocrine tumour, *SCLC* small cell lung carcinoma, *EHE* epithelioid hemangioepithelioma, *ID* study identification number. *a (waterfall)—Of the 49 efficacy-evaluable patients, 3 patients had progressive disease (described in the section “Efficacy evaluation”) but no target lesion measurement (not included in waterfall plot)

At the time of data cut-off, 49 patients (94%) were considered efficacy evaluable: 40 from dose escalation and 9 from dose expansion (Fig. 2a, b). Three of the efficacy-evaluable patients (3/49) had progressive disease as best response without post-baseline measurements of target lesions: 2 with unequivocal progression of non-target lesions, and 1 with investigator assessed clinical progression (pain). The median duration on study for the efficacy-evaluable population was 1.9 months (range 0.1–40.7), and median progression-free survival was 3.2 months (95% confidence interval: 1.6–4.9). Among the efficacy-evaluable patients, 26 (53%) were treated at or above the RP2D.

One PR was observed in a patient with adenoid cystic carcinoma treated at 48 mg who remained on trial (45 cycles) at data cut-off, and 13 patients experienced SD as best response, with 2/13 patients having SD ≥6 months as defined by RECIST v1.1: one with *KRAS*-mutant, microsatellite-stable (MSS) colorectal cancer with a maximum reduction in target lesions of 24% on study for 12 cycles, and a second with MSS endometrial carcinoma (reduction in target lesions of 3.7%) experienced SD for 8 cycles. Progressive disease as best response was observed in 35/49 (71%) patients. No CRs were seen. ORR for the efficacy-evaluable cohort was 2% (1/49), and the CBR was 6% (3/49).

### Correlative studies

Pharmacodynamics could not be formally assessed, as assays for in vivo evaluation of endogenous phosphorylation targets of PLK4 (including phospho-PLK4) were not available. Paired fresh tumour biopsies were obtained in 4 patients enrolled in the 64 mg expansion cohort. Of these, only 2 patients (who remained on study for 2 and 3 cycles) had tumour cells present in both biopsy samples. No treatment-related polyploidy or changes in phospho-histone H3 staining were evident, as shown in preclinical models.^11^ Selected cancer-associated coding mutations identified in archival samples through WES for 6 patients are demonstrated in Fig. 2c. A post-progression tumour biopsy was obtained in one patient with KRAS mutant, MSS colorectal cancer who progressed after 12 cycles at 48 mg. Targeted sequencing of the progression biopsy and exome sequencing of a PDX generated from the biopsy were compared to the archived sample. These analyses did not identify an obvious mediator of acquired resistance; importantly, no kinase gatekeeper mutations in PLK4 or AURKB were present in the post-progression sample.

## Discussion

In this study, we evaluated the first-in-class, selective PLK4 inhibitor CFI-400945 in patients with advanced solid tumours. CFI-400945 was generally well tolerated, with neutropenia as the most common dose-dependent, treatment-related AE. This was predicted by preclinical toxicology studies and is an on-target effect of PLK4 inhibition. Neutropenia was manageable with dose interruptions or reductions, and G-CSF was used in cases of febrile neutropenia with full recovery. No patient-related factors were associated with the development of high-grade neutropenia, and this was almost exclusively associated with higher AUC (>400 ng × h/ml) and *C*_max_ (≥38 ng/ml) (Fig. 1b, c). Although the average *C*_max_ of the 64 mg dose level (44 ng/ml) exceeded this threshold, only 1/15 patients experienced a DLT event. Of note, the patient with febrile neutropenia at 64 mg had rapidly progressive liver disease. As a precaution against early-onset neutropenia, a run-in monitoring period was instituted. While there were no neutropenic events during Cycle 0 in participants enrolled following introduction of this run-in period, the strategy was feasible, safe, and has been incorporated into ongoing phase II studies. Asymptomatic grade 3 lipase and amylase elevation accounted for a DLT at 72 mg. This event occurred in a patient with cholangiocarcinoma who was discovered to be taking a pancreatic enzyme supplement. While no similar AE were observed in other patients, this event was deemed possibly related to CFI-400945 due to its temporal onset. Low-grade treatment-related fatigue and gastrointestinal side effects were common, but these did not impact treatment delivery and may have been related to a heavily pretreated study population.

Evidence of antitumour activity was observed at doses as low as 48 mg, including prolonged SD with target lesion reduction (−24%) in a patient with *KRAS* mutant colorectal cancer. In addition to the patient with endometrial cancer who met the CBR definition of SD (>6 months), a second patient with *AKT1* E17K mutant endometrioid endometrial carcinoma had target lesion reduction (−19%) and remained on study for 6 cycles with SD. Long-term tolerability and activity were shown by prolonged treatment in a patient with adenoid cystic carcinoma treated at 48 mg, who remained on study at the time of data cut-off, with an ongoing PR after 45 cycles.

While the overall efficacy observed was modest in this heavily pretreated population with advanced solid tumours, the absence of toxicities other than myelosuppression is notable and several strategies are being pursued to further develop this first-in-class PLK4 inhibitor. Phase 2 studies in prostate (NCT03385655) and breast cancer (NCT03624543) will evaluate disease-specific cohorts and permit the evaluation of biomarkers of sensitivity in relevant populations. These include PTEN loss, which was identified as a correlate of response to CFI-400945 in preclinical cell line studies^11^ but not identified (by sequencing) in baseline specimens of select patients in this study. Exploration of alternative schedules that may permit achievement of higher peak exposures is of interest based on preclinical studies, and an intermittent schedule of 2 days on, 5 days off is currently under evaluation in dose escalation following an amendment to this study protocol. Based on preclinical data demonstrating combination activity with immune checkpoint blockade in vivo, and the absence of overlapping toxicities with daily dosing, combination studies are in development to evaluate the combination of a PD-L1 immune checkpoint inhibitor with CFI-400945 at the 64 mg daily dose in triple-negative breast cancer.^18^ In addition, the specific effects on myeloid lineages may be relevant to the treatment of haematologic malignancies, and a phase 1 study in patients with refractory acute myeloid leukemia (in whom neutropenia is not an important DLT) is underway (NCT03187288).

## Conclusions

In summary, CFI-400945, a first-in-class inhibitor of PLK4, was tolerable when administered at 64 mg (RP2D) on a continuous dosing schedule in patients with advanced solid tumours. Neutropenia was the principal DLT. The favourable PK characteristics and evidence of preliminary antitumour activity support the further development of this agent and validate PLK4 as a potential therapeutic target in cancer patients.

## Supplementary information

Supplementary Information

## Data Availability

The data sets generated duration and/or analysed during the current study are not publicly available due to patient privacy laws (Personal Information Protection and Electronic Documents Act [PIPEDA]) but are available from the corresponding author on reasonable request.
