# Supplementary material for: Safety and tolerability of CFI-400945, a first-in-class, selective PLK4 inhibitor in advanced solid tumours: a phase 1 dose-escalation trial
Source: Br J Cancer. 2019 Jul 15;121(4):318–24. doi: 10.1038/s41416-019-0517-3 (PMC6738068; doi:10.1038/s41416-019-0517-3)
Supplement: Supplementary file 1 — Supplementary Information [file 41416_2019_517_MOESM1_ESM.docx]

Supplementary information - Additional protocol Information

***1a - patient eligibility criteria information:***

Patients were excluded if they were pregnant or nursing, had received anticancer treatment ≤4 weeks prior to first dose of study drug (with the exception of limited palliative field radiotherapy ≤2 weeks prior to first dose); had unresolved toxicities from prior therapy >grade 1; had clinically significant chronic, active, or acute infections; had uncontrolled severe hypertension; had active or underlying cardiac disorders; gastrointestinal disorders impairing administration or absorption of oral agents or were unable to swallow oral medication; had major surgery ≤21 days prior to starting therapy; had known central nervous system metastases, unless clinically and radiographically stable disease for 3 months and not requiring steroids or anticonvulsants; were taking full dose warfarin or other strong CYP3A4 inhibitors, and had a life expectancy >3 months.

Patients must have had adequate liver, renal, bone marrow, and cardiac function.

***1b - study design and objective information:***

Starting dose of CFI-400945 in humans was based on 1/10^th^ of the severely toxic dose affecting 10% of animals (STD10; rats being the more sensitive species) equating to a fixed dose of 3 mg/day in humans (or 1.8 mg/m^2^ for a BSA of 1.7 m^2^). Three to six evaluable patients per treatment cohort were assigned to receive CFI-400945 continuously in 28-day treatment cycles. Dose escalations occurred in 100% increments for individual dosing cohorts up to 32 mg/day. Subsequent dose escalations continued in increments not exceeding 50%, depending on toxicities experienced, and with the approval of local Investigators and the external Medical Monitor. Dosing levels ranged from 3 mg, 6 mg, 11 mg, 16 mg, 24 mg, 32 mg, 48 mg, 72 mg, and 96 mg in the 3+3 dose-escalation schema.

***1c - MTD, DLT, and RP2D information:***

Hematologic DLTs were defined as drug-related anemia grade ≥3; febrile neutropenia grade ≥3, and/or neutropenia grade ≥3 associated with infection; asymptomatic neutropenia grade ≥3 that persisted for >7 days; asymptomatic thrombocytopenia grade ≥3 that persisted for >7 days following treatment interruption; grade 3 thrombocytopenia associated with bleeding; or grade 4 thrombocytopenia.

Additional DLTs included the occurrence of any drug-related grade 3 non-hematological toxicity despite maximal supportive care; any other grade 4 hematological toxicity; evidence of drug-induced liver injury (ie: AST/ALT >3-fold the upper limit of normal [ULN] with bilirubin >2-fold the ULN; or AST/ALT >3-fold the ULN with evidence of drug-induced hepatitis or hypersensitivity); grade 3 gastrointestinal toxicity (i.e. diarrhea, nausea/vomiting) or asymptomatic electrolyte disturbances persisting >72 hours despite supportive care; cardiac toxicity manifesting as asymptomatic decrease in left ventricular ejection fraction [LVEF] >10% compared to baseline with LVEF below lower limit of normal, confirmed on repeat assessment 7 days after initial documentation, grade 3 troponin (confirmed with repeat testing within 24 hours), or clinical signs of cardiac disease such as unstable angina or myocardial infarction; and grade 3 fatigue lasting >7 days. Dose escalation decisions were made following the completion of Cycle 1 for all patients enrolled in a given dose level, taking into consideration adverse events (AE) and pharmacokinetic results.

***1d - safety assessment information:***

Investigators managed any AE until resolution, stabilization, or until the AE was considered no longer clinically significant. All AE and serious AE (SAE) were recorded in source documents and individual case report forms from the time of first dose of study drug until 30 days following the last dose of study drug.

Safety evaluations were conducted at screening and during Cycle 1 on Day 1, 2, 4, 8, 11, 15, 22, and 28. In the expansion cohort, following the run-in amendment, safety evaluations were conducted on Cycle 0 Day 1, 2, 4, 8, and 11, then as above for Cycle 1. Evaluations included pre-defined combinations of medical history, physical examination, vital signs, clinical laboratory studies, and ECOG performance status.


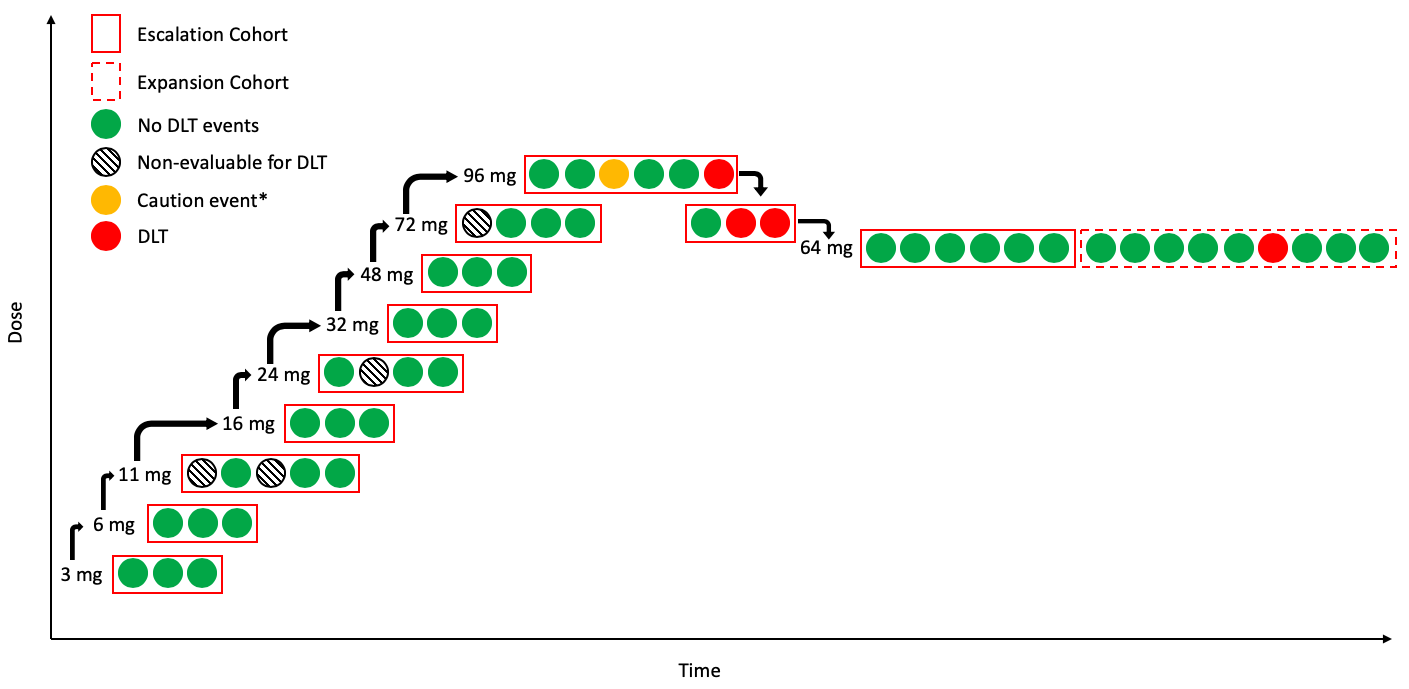
 Supplementary Figure 1**:** Dose-escalation and expansion diagram depicting CFI-400945 dosing levels and patient events.

*Caution event denotes patient who experienced febrile neutropenia event not meeting DLT criteria

*Abbreviations: DLT, Dose Limiting Toxicity*


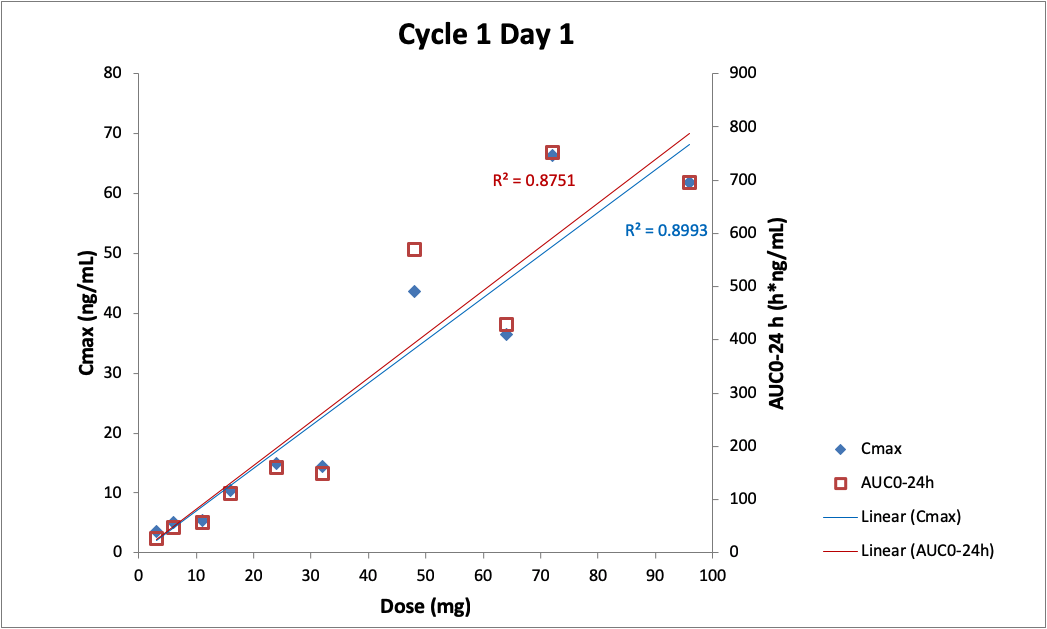
Supplementary Figure 2: Regression analysis of pharmacokinetic relationship between C_max_ and AUC using for varying dose levels on Day 1.

*Abbreviations: AUC, Area under the curve; Cmax, Concentration maximum.*
